# Domination based classification algorithms for the controllability analysis of biological interaction networks

**DOI:** 10.1038/s41598-022-15464-4

**Published:** 2022-07-13

**Authors:** Stephen K. Grady, Faisal N. Abu-Khzam, Ronald D. Hagan, Hesam Shams, Michael A. Langston

**Affiliations:** 1 Graduate School of Genome Science and Technology, University of Tennessee, Knoxville, TN USA; 2 Department of Computer Science and Mathematics, Lebanese American University, Beirut, Lebanon; 3 Department of Electrical Engineering and Computer Science, University of Tennessee, Knoxville, TN USA; 4 Department of Industrial and Systems Engineering, University of Tennessee, Knoxville, TN USA

**Keywords:** Computational biology and bioinformatics, Systems biology, Mathematics and computing

## Abstract

Deciding the size of a minimum dominating set is a classic *NP*-complete problem. It has found increasing utility as the basis for classifying vertices in networks derived from protein–protein, noncoding RNA, metabolic, and other biological interaction data. In this context it can be helpful, for example, to identify those vertices that must be present in any minimum solution. Current classification methods, however, can require solving as many instances as there are vertices, rendering them computationally prohibitive in many applications. In an effort to address this shortcoming, new classification algorithms are derived and tested for efficiency and effectiveness. Results of performance comparisons on real-world biological networks are reported.

## Introduction

Let *G* = <*V,E* > denote a finite, simple, undirected graph of order *n*. A dominating set for *G* is a subset *D* of *V* with the property that every vertex in *V-D* has a neighbor in *D*. A minimum dominating set (MDS) is of course one of smallest cardinality. Its size is usually denoted by γ(*G*). Deciding MDS is both *NP*-complete^1^ and W[2]-complete^2^. It is easy to see that *G* may have as many as 3^*n*/3^ distinct MDS solutions, as is demonstrated by the union of *n*/3 disjoint triangles. A common strategy is therefore to concentrate on significance and classify a vertex as “essential” (aka “critical”) if it is used in every MDS, as “intermittent” if it is used in some but not every MDS, and as “redundant” if it is never used in any MDS.

MDS has found purpose in a wide variety of application domains, spanning topics from network science^3–6^ to sensor placement^7^ to transportation streaming^8^. Compelling utility has been cast in the realm of systems biology, where MDS has been used to model the controllability of biological networks in research fields as diverse as cancer^9–11^, drug discovery^12^, gene regulation^13^, neuroscience^14^, protein interaction^15–17^, and viral infection^18^. Vertex classifications under MDS have even been used in the search for ncRNA’s latent regulatory role in polygenic human disease^19^.

Previous classification strategies examine vertices one by one, and thus invoke an MDS algorithm *n* or more times in the worst case. Efficiency may be achieved in the average case, however, by observing that a vertex is essential should it have two or more pendant vertices^20^ and redundant should all of its neighbors be essential^21^. The main results of this paper generalize and greatly extend these pioneering observations with five novel vertex classification rules with which we can further decrease the number of times MDS must be solved. To accomplish this, we devise highly efficient techniques that can take advantage of neighborhood structure and, if desired, adjacency-preserving vertex permutations. Additionally, we report on experiments conducted over a variety of biological application domain graphs that help demonstrate the relative effectiveness of these innovative new methods.

The remainder of this paper is organized as follows. In the next section, we define some needed notation and briefly review prior work. In a third section, we devise four reduction rules, provide arguments for their soundness, and show how they can be employed to speed the task of vertex classification. In a fourth section, we introduce a fifth rule based on algebraic symmetry and discuss its practical potential. In a fifth section, we evaluate the effectiveness of these inventive rules on a variety of real-world biological problem instances. In a final section, we draw conclusions and consider a few directions for future research.

## Preliminaries

### Notation

Let *u* and *v* denote elements of *V*. The distance between *u* and *v* is the number of edges in a shortest path between them. The neighborhood of *u*, denoted by *N*[*u*], comprises *u* and its neighbors or, equivalently, those vertices within distance one from u. (This is sometimes called the closed neighborhood of *u*, in order to distinguish it from the open neighborhood *N*[*u*] − {*u*}.) Neighborhoods are extended to sets in a straightforward fashion. Thus, for a set *S* of vertices, *N*[*S*] denotes *S* and the neighbors of all its elements. An orbit is an equivalence class of vertices under the action of an automorphism group. That is, *u* and *v* belong to the same orbit if and only if there exists a relabeling of *V* that results in an isomorphic graph for which *u* and *v* have exchanged labels^22^. Finally, given an MDS, *D*, we say that *u* dominates *v* whenever *u* and *v* are adjacent, and *u* but not *v* is an element of D.

### Prior work

The vertex classification problem has been studied^20,21^ using the two previously-mentioned observations coupled with an MDS algorithm that employs Integer Linear Programming (ILP). Despite the fact that known ILP methods can in principle require exponential time, a major appeal of this approach relies on the existence of powerful commercial ILP solvers that tend to work extremely well in practice. Thus, once an initial MDS, *D*, has been computed, one needs only to consider each vertex, *u*, in turn.

- If *u* ∈ *D*, then construct an ILP instance of MDS with a constraint to exclude *u*. We refer to the resultant procedure as ILP-exclude, with parameters *G* and *u*. If γ(ILP-exclude(*G,u*)) exceeds γ(*G*), then *u* is essential, otherwise it is intermittent.
- And if* u* ∉ *D*, then construct an ILP instance of MDS with a constraint to include *u*. We refer to the resultant procedure as ILP-include, also with parameters *G* and *u*. If γ(ILP-include(*G,u*)) exceeds γ(*G*), then *u* is redundant, otherwise it is intermittent.

### Classifier A

For the sake of clarity and exposition, and to help explicate algorithmic comparisons, this procedure (previously unnamed in^20,21^) is presented here in pidgin code and dubbed Classifier A. We note that the exploitation of pendant vertices can be employed at start-up, while the examination of neighbors is best applied only after all essential vertices have been identified.
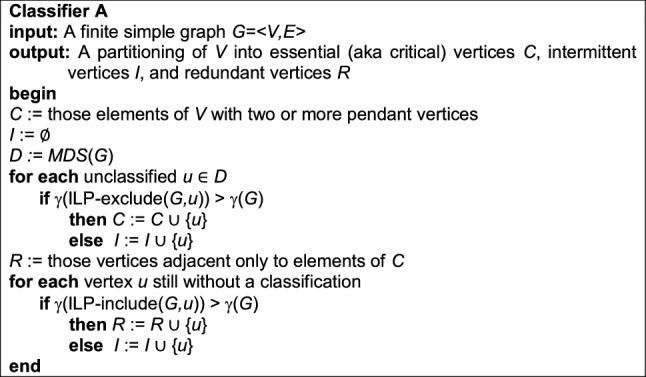


Classifier A requires low-order polynomial time to initialize *C* and *R* (an exact upper bound depends on graph density and the data structures used), exponential time for a call to an ILP solver to answer a single instance of MDS, and time for at most *n* exponential-time calls to ILP-exclude/include. Classifier A’s needs for extra space are negligible.

## In search of a better classifier

### Classification rules

Classifier A’s most time-consuming operations are its multitude of calls to ILP-exclude/include. We therefore propose, scrutinize, and employ a series of pre-processing rules in hopes that we can reduce the total number of calls required, thereby increasing the scalability of MDS-based biological network analytics.

**Rule 1.** Suppose *u* and *v* are adjacent, and the neighborhood of *u* is a proper subset of the neighborhood of *v*. If *v* is essential, then *u* is redundant.

Soundness. If an MDS contains *v*, then it cannot contain *u*, since otherwise the MDS would not be minimum. Thus, if every MDS contains *v*, then none can contain *u*. (Note the need for proper containment. If *N*[*u*] = *N*[v], then neither *u* nor *v* can be essential, and both must be redundant or both intermittent.)

**Rule 2.** If *u* is not essential, and if every element in *u*’s neighborhood is either essential or adjacent to an essential vertex, then *u* is redundant.

Soundness. This is a generalization of Rule 1, in which vertices in the neighborhood of *u* may be dominated by more than just a single essential vertex.

**Rule 3.** Suppose *u* but not *v* is contained in an MDS for which those vertices dominated only by *u* are in the neighborhood of *v*. Then both *u* and *v* are intermittent.

Soundness. Replacing *u* with *v* produces a distinct but equivalent MDS.

**Rule 4.** If *u* has neighbors *v* and *w* whose only common neighbor is *u* and for which (*N*[*N*[*v*]] ∪ *N*[*N*[*w*]]) ⊂ *N*[*u*], then *u* is essential.

Soundness. Because *N*[*v*] ∩ *N*[*w*] = {*u*}, and because *u* dominates every vertex in *N*[*N*[*v*]] ∪ *N*[*N*[*w*]], it follows that *u* is required in any MDS, since otherwise at least two vertices from *N*[*v*] ∪ *N*[*w*] would be required in its place to dominate *v* and *w*.

**Figure 1:**
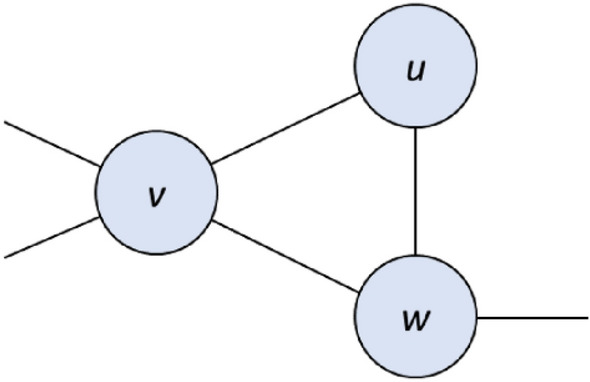
A sample subgraph subject to Rule 1. If *v* is essential, then *u* is redundant.

**Figure 2:**
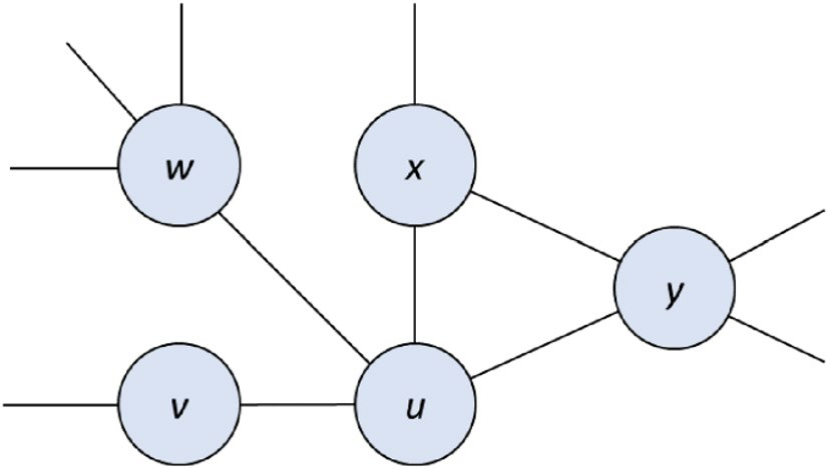
A sample subgraph subject to Rule 2. If every element in the set {*u, v, w, x, y*} is either essential or adjacent to an essential vertex, then *u* is either essential or redundant.

**Figure 3:**
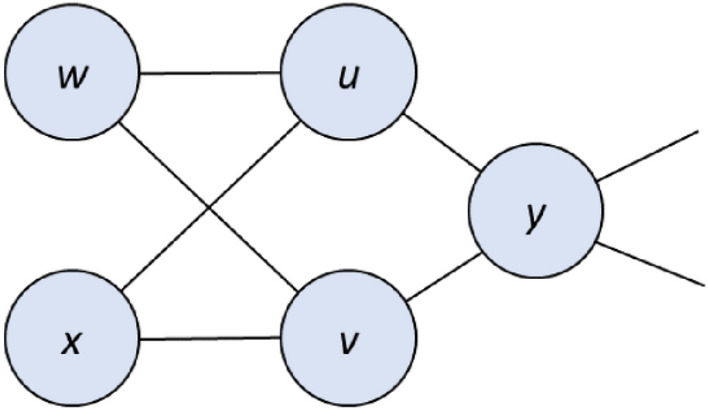
A sample subgraph subject to Rule 3. If *u* but not *v* is contained in an MDS, then *v* but not *u* is contained in some other MDS, and so both vertices are intermittent.

**Figure 4:**
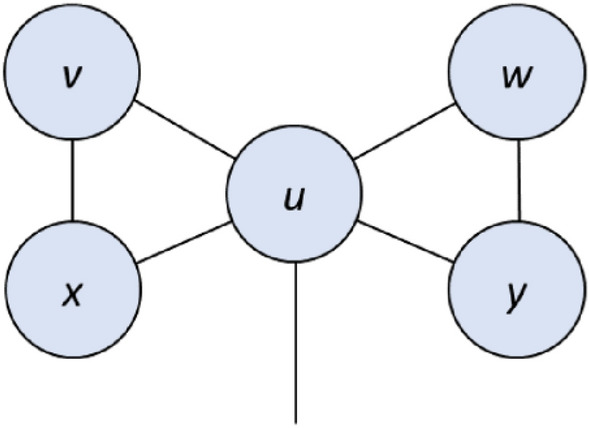
A sample subgraph subject to Rule 4. Vertex *u* must be essential.

These rules require only neighborhood explorations, and are thus amenable to illustration. Sample subgraph configurations are depicted in Figs. 1, 2, 3 and 4.

### Classifier B

We make use of these four rules in a procedure we name Classifier B. This new classifier need not invoke Classifier A, because the aforementioned observations upon which Classifier A relies are subsumed by Rules 2 and 4. On the other hand, the order in which rules are applied by Classifier B is important if we are to avoid calling MDS multiple times.
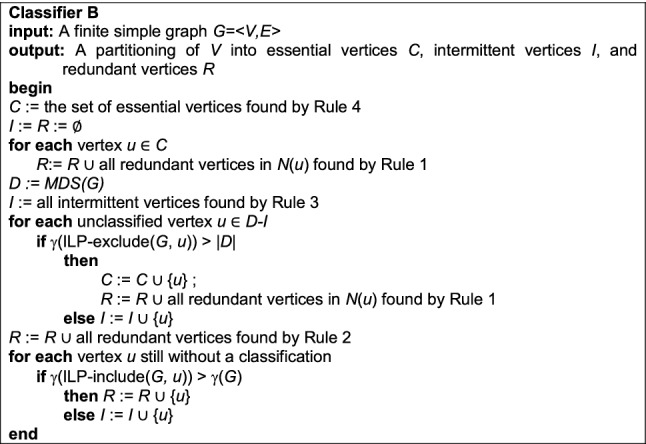


Classifier B’s resource requirements are similar to those of Classifier A. It needs low-order polynomial time to apply Rules 1–4 in the computation of *C*, *I* and *R* (an exact upper bound again depends on graph density and the data structures used), exponential time for a call to an ILP solver to answer a single instance of MDS, and time for at most *n* exponential-time calls to ILP-exclude/include. Classifier B’s needs for extra space are negligible.

Bolstered by Rules 1–4, it should come as no surprise that Classifier B provides a considerable improvement over Classifier A. We will demonstrate this convincingly in the sequel. But first we consider the possible utility of a more computationally demanding rule.

## The use of algebraic symmetry

### Orbits and automorphisms

In an effort to provide additional reductions in the number of ILP-exclude/include calls required, we turn to notions of graph structure, neighborhood symmetry, and adjacency-preserving vertex permutations.

**Rule 5.** If *V* is partitioned into a set of vertex orbits, then vertices within the same orbit must possess the same classification.

Soundness. Vertices within the same orbit are indistinguishable under automorphic transformation, and so their classifications will be identical.

### Classifier C

We therefore study yet a third procedure, which we christen Classifier C. This new classifier operates as does Classifier B, except that it incorporates Rule 5 by first computing all orbits and then, whenever a vertex is classified, any unclassified vertices in its orbit are assigned the same classification.

Classifier C, like Classifier B, requires low-order polynomial time to apply Rules 1–4, exponential time to solve a single instance of MDS, and time for at most *n* exponential-time calls to ILP-exclude/include. Classifier C also needs low-order polynomial time to update orbit classifications. More significantly, it requires exponential time to determine the orbits themselves with known practical methods^23^. These orbits can be found using bliss^24^, nauty^25^, and a variety of other popular, well documented, easy-to-use tools. From these we chose saucy^26,27^, by virtue of the fact that it has been tuned for sparse graphs, which are overwhelmingly representative of large-scale biological data. And indeed, saucy was roughly 10–20 times faster than bliss and over 1000 times faster than nauty across our test suite. We hasten to add, however, that saucy requires a bit more effort to implement than does nauty or bliss. This is because saucy only returns vertex pairs that occupy the same orbit. The user must then merge these pairs to form a complete orbit set. Classifier C’s needs for extra space are negligible.

## Classifier comparisons

### Computational milieu

Classifiers A, B, and C were implemented in C+ + and compiled using the g+ + (GCC) version 4.8.5 compiler under the CentOS Linux 7 × 86–64 operating system. Various mathematical optimization software packages were considered, including notable options such as CPLEX^28^ and Xpress^29^. From these we chose Gurobi^30^ for our ILP solver. It is a hugely successful, widely used, state-of-the-art commercial product. Moreover, Gurobi is freely available to many in the research community via academic site license. As in previous work, we used ILP to satisfy each classifier’s initial MDS requirement. Possible alternatives include the measure and conquer method of^31^, which runs in *O*(1.4864^*n*^) time and polynomial space. We were careful to avoid reproducibility problems that might arise from complex parameter settings. Our classifiers take as input only finite simple graphs, while default settings were strictly obeyed for Gurobi.

In order to provide empirical comparisons at scale, all tests were executed on the Advanced Computing Facility (ACF) computational cluster maintained by the National Institute for Computational Sciences^32^. Timings were performed on a single core of ACF’s monster (big memory) node using a Dell PowerEdge R630 server, an Intel Xeon E5-2687 W v4 30 MB Intel Smart Cache 3.00 GHz processor, 1,024 GB DDR4 memory, and ACF's read/write Network File System.

Three dozen challenging graphs were assembled to form a comprehensive classifier test suite. Graphs that populate this suite were obtained from well-known repositories and derived from transcriptomic, proteomic, epigenetic, and a variety of other sorts of biological data. We excluded from this suite any graph on which a classifier failed to finish within 24 h, which generally seemed to result from exceptional size or, less frequently, from unusual density. Graphs thusly selected are described in Table 1. Runtimes per instance and classifier are displayed in Table 2.

**Table 1:** A test suite of real-world biological graphs.

Index	Graph name	Type	Source(s)	|V|	|E|	γ
1	HiC-Net-1	CI	^37^	1099	32,848	17
2	HiC-Net-3	CI	^37^	1084	31,724	19
3	HiC-Net-5	CI	^37^	1419	43,763	25
4	HiC-Net-7	CI	^37^	1083	32,336	18
5	HiC-Net-10	CI	^37^	1094	30,216	20
6	HiC-Net-11	CI	^37^	1165	38,784	17
7	HiC-Net-14	CI	^37^	1056	33,851	15
8	HiC-Net-15	CI	^37^	1164	35,470	19
9	HiC-Net-21	CI	^37^	1376	41,314	22
10	GIANT-top-brain-02-filtered	GC	^38^	14,306	1,358,435	1159
11	Pancreas_GDS4102_control.995	GC	^39–42^	2591	61,245	650
12	ProteomeHD-top-05-co-regulated	GC	^38^	2717	62,749	505
13	ColorectalCancer_GSE9348_control.975	GC	^39,43^	2803	3918	1099
14	BreastCancer_GSE10810_case	GC	^39,44^	3249	7070	1197
15	ParkinsonsDisease_GSE20141_case.996	GC	^39,45^	2340	12,959	738
16	cerebellum-male	GC	^46^	10,274	78,981	2605
17	yeast-8	GC	^39,47^	5544	389,058	409
18	bio-CE-GT	GFA	^48^	924	3239	126
19	bio-CE-GN	GFA	^48^	2220	53,683	195
20	Bio-HS-HT	GFA	^48^	2570	13,691	456
21	BioGrid-PP-Interaction-A-thaliana	PPI	^38^	10,823	51,278	1353
22	Y2H-union	PPI	^49^	1966	2705	575
23	bio-grid-fission-yeast	PPI	^48^	2026	12,637	280
24	HC-BIOGRID-2.0.31	PPI	^50^	2538	6418	607
25	bio-grid-worm	PPI	^48^	3507	6531	578
26	HuRi	PPI	^51^	8275	52,088	1341
27	bio-grid-fruitfly	PPI	^48^	7274	24,894	1522
28	bio-wormnet-v3	PPI	^48^	16,347	762,822	2072
29	bio-grid-human	PPI	^48^	9436	31,182	1785
30	PP-Decagon-ppi	PPI	^37^	19,081	715,612	1353
31	Lit-BM	PPI	^38^	5956	12,758	1322
32	FF-miner-miner-func-func	M	^38^	46,027	106,510	6751
33	ChCh-Miner-drugbank-chem-chem	M	^48^	1514	48,514	93
34	NCI-PID-complete-interactions	M	^48^	2855	25,433	247
35	bn-fly-drosophila-medulla-1	M	^37^	1781	8911	317
36	bn-mouse-retina-1	M	^37^	1076	90,811	14

Types are CI (chromatin interaction), GC (gene co-expression), GFA (gene functional association), PPI (protein–protein interaction), and M (miscellaneous), where graph 32 is derived from biological functionality data, graph 33 is derived from drug-drug interactions, graph 34 is derived from human gene signaling and regulatory pathway interactions, and graphs 35 and 36 are derived from neuron connections in the fly medulla and in the mouse retina, respectively.

**Table 2:** Run times for each test suite instance and each classifier, measured in seconds.

Index	Graph name	Classifier A	Classifier B	Classifier C
1	HiC-Net-1	18.47	15.37	7.586
2	HiC-Net-3	15.054	8.689	6.571
3	HiC-Net-5	36.248	23.372	13.646
4	HiC-Net-7	30.824	6.833	4.65
5	HiC-Net-10	17.334	8.126	6.955
6	HiC-Net-11	19.584	8.698	6.342
7	HiC-Net-14	21.723	7.265	3.852
8	HiC-Net-15	16.313	6.589	4.231
9	HiC-Net-21	48.166	25.792	18.477
10	GIANT-top-brain-02-filtered	1.628	0.394	0.441
11	Pancreas_GDS4102_control.995	37.308	23.598	17.987
12	ProteomeHD-top-05-co-regulated	16.162	6.238	5.109
13	ColorectalCancer_GSE9348_control.975	9903.397	3854.583	3499.532
14	BreastCancer_GSE10810_case	42.488	17.14	13.543
15	ParkinsonsDisease_GSE20141_case.996	62.139	25.381	14.327
16	cerebellum-male	11.396	2.567	3.708
17	yeast-8	17.793	2.542	4.848
18	bio-CE-GT	45.532	27.24	16.747
19	bio-CE-GN	317.515	105.023	165.61
20	Bio-HS-HT	976.197	458.888	341.572
21	BioGrid-PP-Interaction-A-thaliana	151.596	26.463	43.407
22	Y2H-union	3.216	0.591	1.999
23	bio-grid-fission-yeast	11.455	1.684	1.684
24	HC-BIOGRID-2.0.31	11.074	3.146	3.417
25	bio-grid-worm	4.856	1.056	1.842
26	HuRi	115.966	20.366	33.67
27	bio-grid-fruitfly	80.533	22.184	27.024
28	bio-wormnet-v3	8533.929	4352.012	4791.11
29	bio-grid-human	118.688	27.055	46.685
30	PP-Decagon-ppi	10,369.653	6483.164	5656.752
31	Lit-BM	37.214	7.03	13.032
32	FF-miner-miner-func-func	21.408	7.394	8.435
33	ChCh-Miner-drugbank-chem-chem	42.936	27.369	33.892
34	NCI-PID-complete-interactions	5.955	0.961	3.518
35	bn-fly-drosophila-medulla-1	25.876	10.465	7.175
36	bn-mouse-retina-1	8728.462	3226.134	4704.256

### Empirical results

**Figure 5:**
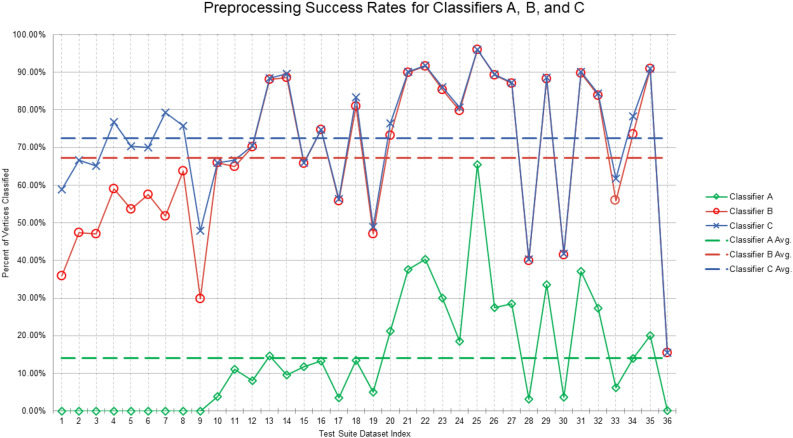
Percent of vertices classified without ILP-exclude/include calls by Classifiers A (in green), B (in red), and C (in blue). Dashed lines represent averages, which were 14.1%, 67.2%, and 72.5% for Classifiers A, B, and C, respectively.

We first studied preprocessing, with success measured as a percentage of vertices classified without an ILP-exclude/include call. Over our test suite, Classifier A had an average success rate of only 14.1%. In contrast, Classifier B had an average success rate of 67.2%, while Classifier C had an average success rate of 72.5%. As expected, Rules 1–5 thus seem to place Classifiers B and C at an enormous computational advantage. See Fig. 5.

**Figure 6:**
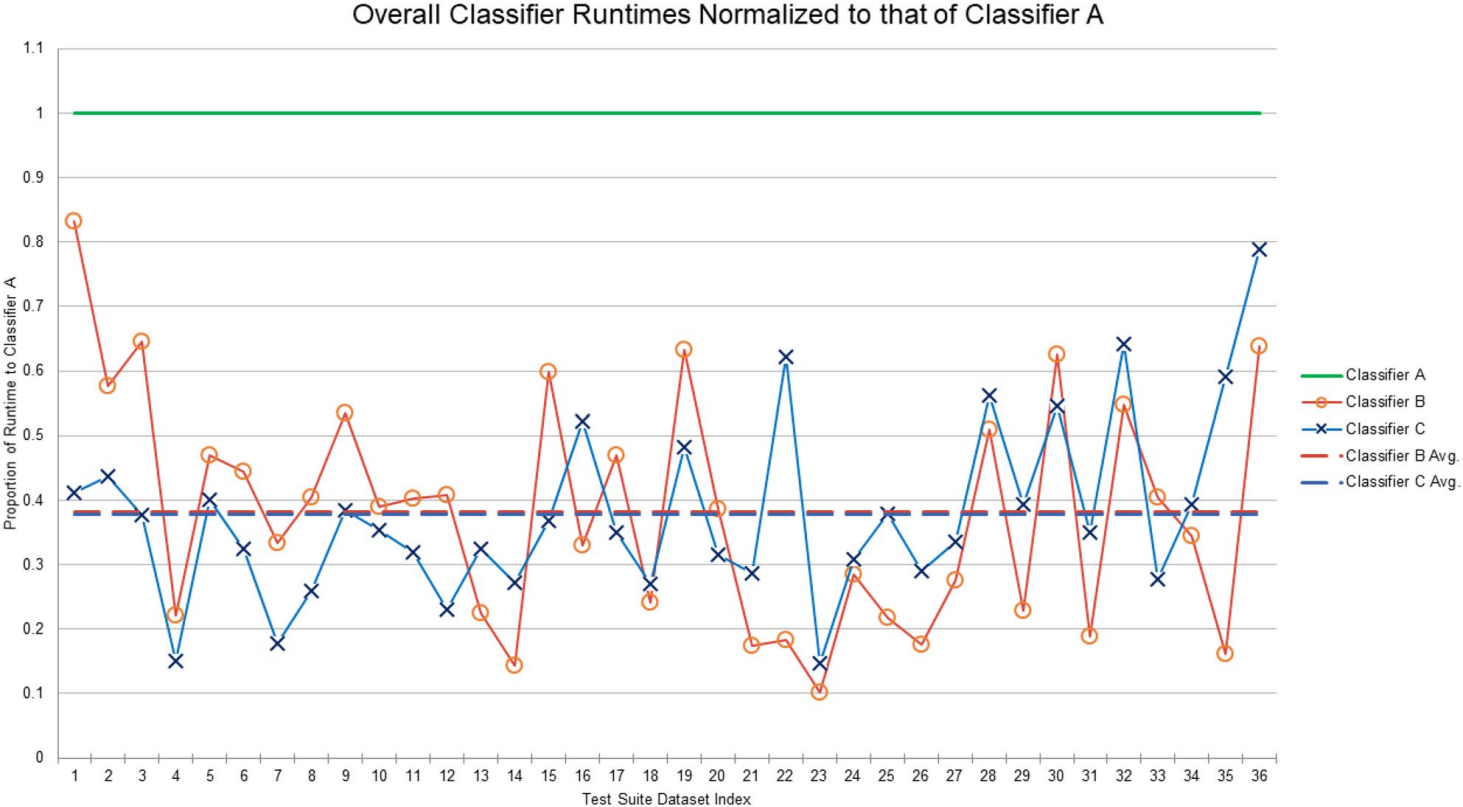
Overall runtimes of Classifiers B (in red) and C (in blue), normalized to that of Classifier A (in green). Dashed lines are almost collinear and represent averages, which were 38.2% and 37.9% for Classifiers B and C, respectively.

We then turned to overall processing times. Unsurprisingly, we found that Classifier A was simply not competitive. Its meager preprocessing success rate placed too great a burden on mathematical optimization software. The computational demands of Rule 5, however, posed a pivotal question: is Classifier C’s modest reduction in ILP-exclude/include invocations a smart investment? In other words, do Classifier C’s time-consuming orbit computations translate into runtimes that are better than those of Classifier B? The answer is hardly obvious. Even with a leading-edge graph automorphism package such as saucy, it can be exceedingly difficult to compete against ILP computations performed by a well-honed commercial product like Gurobi. Because runtimes varied greatly over the graphs in our test suite, we normalized all completion times to that of Classifier A. Resultant calculations revealed that, on average, Classifiers B and C were more or less in a dead heat. Classifier B took roughly 38.2% as long as Classifier A, while Classifier C took some 37.9% as long. Thus, under these experimental conditions, the overall impact made by adding Rule 5 was positive but barely noticeable. See Fig. 6.

It is difficult from these results to argue against the use of either Classifier B or Classifier C. Both are vastly more effective than Classifier A. And while Classifier B is the simpler of the two, Classifier C was able to eke out a slight gain in speed. Having said that, we must remember that this endorsement is dependent on both our test suite and the computational resources available. Classifiers B and C were highly competitive. Different datasets, alternative applications, or a change in automorphism software may cause the added overhead and complexity of Classifier C to have a much greater effect, either positive or negative, than was observed here. These experiments in fact prompt a few serendipitous dataset observations, which we will discuss in the final section.

## Discussion

### Conclusions

Major contributions of this paper include the development, analysis, implementation, and testing of five novel classification rules and two highly innovative classifier algorithms with which vertex significance can be gauged in a network domination setting. Extensive empirical evidence of the practical usefulness of these powerful new rules and classifiers was also generated using a comprehensive test suite centering on life science applications and biological data.

Classifiers B and C turn out to be huge improvements over Classifier A in terms of both preprocessing rates and overall runtimes. Their relative effectiveness would have been even more pronounced had we not had access to a commercial ILP solver with the exceptional efficiency of Gurobi. Results from our extensive test suite suggest that Classifiers B and C are very nearly equal in performance. Although Classifier C was faster by a narrow margin, users may wish to give Classifier B a slight nod for its comparative simplicity.

Patterns seen in results and data may be of additional interest. We observe, for example, the modest MDS size of chromatin interaction data (test graphs 1–9). Concomitantly, these are the only graphs for which the preprocessing performed by Classifier C is significantly better than that of Classifier B. It seems plausible that this rather curious situation might be attributable to graph density, but most biological data is sparse, and indeed these graphs are roughly as sparse as all others in our test suite. We therefore turned to degree distributions and found that the chromatin interaction histograms appear normalesque and not scale-free like histograms for the rest of our test suite. Whether this is causative is unknown. We found it interesting too that all classifiers were unusually successful in preprocessing graph 25 (bio-grid-worm). Upon investigation, we discovered that this graph has an extremely high number of redundant vertices. Whether this attribute relates to better preprocessing is unclear. And finally, graph 36 (bn-mouse-retina-1) caught our attention because it was especially difficult for all classifiers, and yet its MDS is about the same size as those of the chromatin interaction graphs. Other than idiosyncrasies of data capture (neuronal connections imaged by electron microscopy), we can posit no particular basis for its computational recalcitrance.

### Directions for future research

The rules we have devised assign a single MDS classification to any vertex. It is sometimes possible, however, to eliminate one classification option, making it reasonable to envisage more convoluted rules that assign a pair of classification choices to some vertices. As we have seen with Rule 5, however, the overhead and complexity of such a strategy must not be so high that it negates any meaningful gains.

MDS vertex classifications may find additional utility among problem variants. The study of independent dominating set, for instance, is a restatement of maximal independent set, and can be traced back roughly 60 years^33^. Other classic examples include connected dominating set^34^ and total dominating set^35^. Vertex classification strategies may also be of interest when data is drawn from reduced graph families. Limiting inputs to planar graphs, for example, is a popular restriction in circuit layout and many other engineering applications, although in our opinion this sort of limitation would be difficult to motivate from a biological perspective.

It might also be instructive to consider the relationship between orbit distributions and graph structure. For example, those who embrace the once-popular scale-free hypothesis^36^ might predict that orbits would be found primarily among leaves that share a common neighbor. As a simple test, we therefore scanned the non-singleton orbit lists and computed the percentage of these lists that contained non-leaf vertices for each graph in our test suite. These values turned out to range more or less uniformly between 4 and 100%. Unsurprisingly, it thus appears that the utility of automorphic transformation is highly data dependent, and that the extent to which Rule 5 applies is primarily a function of the particular graph under examination. This would seem to suggest that the relationship between orbits and the topology of graphs derived from biological data might warrant future study.

Finally, while our focus has been on practical applications, numerous theoretical questions beckon. We think it highly probable, for example, that classification strategies such as those we have developed here may prove useful for combinatorial problems other than MDS. Rule 5, in particular, seems to have something of a universal appeal. Another good example rests with worst-case classifier behavior. Each method we have considered could in principle invoke an MDS solver as many as *n* + 1 times. Classifier A in fact did exactly this, for instance, on test graph 5 (HiC-Net-10). Classifiers B and C, on the other hand, never even came close to this sort of pathology. We think it is highly unlikely that real-world biological data of sufficient size would cause either of these classifiers to be so completely ineffective. To the best of our knowledge, however, the sort of worst-case performance that might be attained with highly contrived data remains unknown.

## Data Availability

Software and data produced as part of this study are available at the following github repository: https://github.com/sgrady3/MDS-vertex-classification.
